# Sickness absence and disability pension patterns before and after ischemic stroke: A Swedish longitudinal cohort study with matched references

**DOI:** 10.1177/23969873241261011

**Published:** 2024-06-14

**Authors:** Mihae Roland, Ann-Sofie Rudberg, Kristina Alexanderson, Christina Sjöstrand

**Affiliations:** 1 Department of Clinical Neuroscience, Karolinska Institutet, Stockholm, Sweden; 2 Department of Clinical Sciences, Danderyd Hospital, Karolinska Institutet, Stockholm, Sweden

**Keywords:** Ischemic stroke, socioeconomic factors, sick leave, working age, cohort study

## Abstract

**Introduction:**

The aim was to determine ischemic stroke patients’ sickness absence and disability pension before and after stroke, and compare these to that of matched references.

**Patients and Methods:**

All working-aged individuals (aged 18–61) in Sweden with incident ischemic stroke in year 2000, 2005, 2010, or 2015, respectively, and five population-based matched references to each stroke patient. Each cohort was followed 1 year prior stroke and 3 years after. We calculated rates and mean days of sickness absence and disability pension among stroke patients and references and computed trajectories of absence days with predictors of high sickness absence and disability pension.

**Results:**

Number of patients with incident ischemic stroke in 2000 (*N* = 2728), 2005 (*N* = 2738), 2010 (*N* = 2767), and 2015 (*N* = 2531). Mean stroke age was 53 years and rate of men was 64%. Mortality rate within 12 months after stroke date decreased from 8.1% in 2000 to 4.8% in 2015. Sickness absence for patients was 31.1% in the year prior their stroke, versus 13.7% for references, both groups mainly due to mental and musculoskeletal diagnoses. Factors associated with future high mean number of sickness absence and disability pension days were elementary educational level; adjusted OR (CI) 3.47(2.38–5.05), being single; 1.67(1.29–2.16), female sex 1.72(1.31–2.26), diabetes; 1.86(1.18–2.92), and aged >50; 2.25(1.69–2.98).

**Discussion and Conclusion:**

Ischemic stroke patients have more absence days compared to matched references even before the stroke, mainly related to mental and musculoskeletal diagnoses. Future research should address the impact of efficient stroke treatment on sickness absence and disability pension.

## Graphical abstract

**Graphical abstract:**
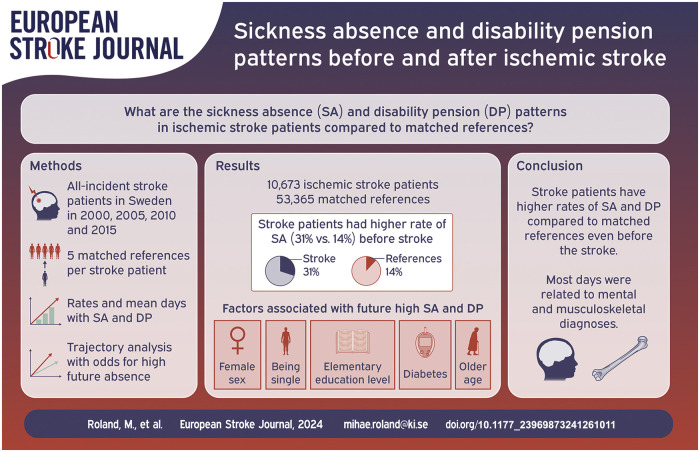

## Introduction

Stroke in working ages is particularly devastating, leading to severe implications for individuals, employers, and society.^1^ Previous studies show that about 30% of stroke patients were on sickness absence, and 20–25% were on disability pension before stroke.^2,3^ Stroke patients accumulate days of sickness absence and/or disability pension already before the stroke event.^1,4,5^ However, only one study (*N* = 6637), separated stroke of ischemic and hemorrhagic origin, an important distinction for the possibility of effective treatment in the acute stage as well as regarding the prognosis.^1^ Stroke affects populations with low socioeconomic status disproportionally in terms of both risk of stroke and outcome.^6–8^ Consequently, it could be suggested that socioeconomic status is an important factor regarding patients’ length of sickness absence and disability pension, motivating the use of matched references from the general population when making such comparisons. Therefore, we performed a nationwide study on previous and future sickness absence and disability pension among people with incident ischemic stroke, during different timeframes and compared to population-based matched references without previous stroke.

The main aim was to determine sickness absence and disability pension rates and days before and after ischemic stroke, compare such rates and days to those among matched references from the general population. Secondary aim was to compute trajectories of annual sickness absence and disability pension days among stroke patients and to identify predictors for future high such numbers.

## Patients and methods

Four cohorts of incident ischemic stroke patients and matched references were studied, each cohort was followed over 4 years.

### Study population and data sources

Anonymized microdata, linked at individual level by use of the unique personal identification number given all residents in Sweden,^9^ from the following four nationwide registers were used:

- The in-patient register, kept by the Swedish Board of Health and Welfare, for the years 1969–2016, regarding main diagnoses for hospitalization, based on ICD-10^10^ code I63, ICD-9 codes 433 and 434, and ICD-8 codes 432, 433, and 444. Information about hypertension (I10.9), atrial fibrillation (I48), and diabetes (E10, E11) as comorbidity diagnoses at the stroke event was also obtained. This register was used for identification of patients, for exclusion of people with previous stroke in possible references, and for the stroke date, defined as the date of hospitalization due to the ischemic stroke.
- The Longitudinal Integration Database for Health Insurance and Labor Market Studies (LISA), kept by Statistics Sweden, to identify patients and matched references living in Sweden, and for information on sociodemographics (sex, age, birth country, type of living area (based on population density),^11^ family situation, marital status, educational level, and emigration). For categorization of these factors, see Table 1. Data was missing on birth country for one individual in the 2005 cohort and two individuals in 2010; those were categorized as “Rest of the World.” Information on educational level was missing for 199 (1.5%), 175 (1.1%), 165 (1.0%), 171 (1.1%) patients in the 2000, 2005, 2010, and 2015-cohorts, respectively; those were categorized as having the lowest educational level.
-The Cause of Death Register, kept by the Board of Health and Welfare, regarding date of death.
- The Micro-Data for Analysis of the Social Insurance register (MiDAS), kept by the Swedish Social Insurance Agency, regarding sickness absence in spells exceeding 14 days and disability pension (dates, diagnoses, and extent) in 1999–2019.

**Table 1.** Baseline characteristics of ischemic stroke patients and matched references for index year 2000, 2005, 2010, and 2015, respectively.

Ischemic stroke incidence year	2000		2005		2010		2015	
	Patients	References	Patients	References	Patients	References	Patients	References
*n*	2728	13,640	2738	13,690	2676	13,380	2531	12,655
Age (mean years (SD))	53.44 (7.38)	53.44 (7.38)	53.97 (7.35)	53.97 (7.35)	52.69 (7.96)	52.69 (7.95)	52.24 (8.23)	52.24 (8.23)
Men (%)	1769 (64.8)	8845 (64.8)	1762 (64.4)	8810 (64.4)	1733 (64.8)	8665 (64.8)	1608 (63.5)	8040 (63.5)
Birth country (%)								
Sweden	2309 (84.6)	11,545 (84.6)	2316 (84.6)	11,580 (84.6)	2181 (81.5)	10,905 (81.5)	2044 (80.8)	10,220 (80.8)
Nordic countries (excl. Sweden)	195 (7.1)	975 (7.1)	188 (6.9)	940 (6.9)	158 (5.9)	790 (5.9)	111 (4.4)	555 (4.4)
Europe (excl. Nordic countries)	140 (5.1)	700 (5.1)	132 (4.8)	660 (4.8)	180 (6.7)	900 (6.7)	176 (7.0)	880 (7.0)
Rest of the world (incl. missing,^*^)	84 (3.1)	420 (3.1)	102 (3.7)	510 (3.7)	157 (5.9)	785 (5.9)	200 (7.9)	1000 (7.9)
Married or civil partnership (%)	1444 (52.9)	8346 (61.2)	1319 (48.2)	7960 (58.1)	1241 (46.4)	7219 (54.0)	1060 (41.9)	6334 (50.1)
Educational level (%)								
Elementary (⩽9 years) (incl. missing^†^)	1088 (39.9)	5440 (39.9)	877 (32.0)	4385 (32.0)	688 (25.7)	3440 (25.7)	584 (23.1)	2920 (23.1)
High school (10–12 years)	1184 (43.4)	5920 (43.4)	1318 (48.1)	6590 (48.1)	1340 (50.1)	6700 (50.1)	1283 (50.7)	6415 (50.7)
University/college (>12 years)	456 (16.7)	2280 (16.7)	543 (19.8)	2715 (19.8)	648 (24.2)	3240 (24.2)	664 (26.2)	3320 (26.2)
Family situation (%)								
Married/cohabitant without children^‡^	1154 (42.3)	6566 (48.1)	1048 (38.3)	6270 (45.8)	950 (35.5)	5289 (39.5)	774 (30.6)	4700 (37.1)
Married/cohabitant with children	358 (13.1)	2205 (16.2)	375 (13.7)	2311 (16.9)	467 (17.5)	2809 (21.0)	445 (17.6)	2616 (20.7)
Single without children	1150 (42.2)	4592 (33.7)	1236 (45.1)	4765 (34.8)	1165 (43.5)	4856 (36.3)	1212 (47.9)	4909 (38.8)
Single with children	66 (2.4)	277 (2.0)	79 (2.9)	344 (2.5)	94 (3.5)	426 (3.2)	100 (4.0)	430 (3.4)
Type of living area (%)								
Cities	927 (34.0)	4635 (34.0)	933 (34.1)	4665 (34.1)	914 (34.2)	4570 (34.2)	855 (33.8)	4275 (33.8)
Towns and suburbs	1167 (42.8)	5835 (42.8)	1154 (42.1)	5770 (42.1)	1187 (44.4)	5935 (44.4)	1090 (43.1)	5450 (43.1)
Rural areas	634 (23.2)	3170 (23.2)	651 (23.8)	3255 (23.8)	575 (21.5)	2875 (21.5)	586 (23.2)	2930 (23.2)
Hypertension (%)	516 (19.2)	..	800 (29.5)	..	1084 (40.7)	..	1005 (40.0)	..
Atrial fibrillation (%)	125 (4.6)	..	150 (5.5)	..	177 (6.6)	..	182 (7.2)	..
Diabetes (%)	333 (12.4)	..	413 (15.2)	..	375 (14.1)	..	369 (14.7)	..

* Missing each year: 0, 1, 2, 0, respectively.

† Missing each year: 199, 175, 165, 171, respectively.

‡ Children <18 years, living at home.

Index year (the year of the stroke) for the different four cohorts was defined as: 2000, 2005, 2010, and 2015, respectively. We included all people living in Sweden and admitted to hospital during the respective index year for a first ischemic stroke diagnosis (ICD-10: I63:0-9) as main diagnosis, when aged 18–61 years. Individuals with prior stroke event in 1969–2015 were excluded. For each included stroke patient, we included five randomly chosen reference individuals from the population with no prior stroke, matched on index year and the five parameters; age, sex, birth country, educational level, and type of living area.

For each patient and reference we obtained sickness absence and disability pension data in the year (365 days) prior the date of the stroke event (Y_-1_) and in the 3 years after the stroke date (Y_1_, Y_2_, Y_3_), as well as on death and emigration.

### Sickness absence insurance in Sweden

Residents in Sweden ⩾16 years, with income from work (including self-employed) or unemployment benefits can claim sickness absence benefits if their work capacity is reduced due to morbidity. Employers provide sick pay for the first 14 days of a sickness absence spell, thereafter, the Social Insurance Agency provides. Thus, all spells included in the analyses had lasted for at least 15 days. There is no limitation to sickness absence spells duration, they can go on for years. People aged 19–64 can be granted disability pension if their work capacity is reduced long-term due to morbidity. Sickness absence and disability pension can be granted for full- or part-time (75%, 50%, or 25%) of ordinary work hours. This means that people can be on part-time sickness absence and disability pension at the same time. Therefore, we computed net days, for example, two gross absence days for 50% were counted as one net days. Only net days are presented below. Sickness absence benefits cover 80% and disability pension about 64% of lost income, up to a certain level.^12^

### Statistical analysis

We computed descriptive statistics regarding the socioeconomic factors, the rates with sickness absence and disability pension, as well as mean annual sickness absence/disability pension days for stroke patients and references in each of the four cohorts.

To explore trajectories of sickness absence and disability pension days per year among stroke patients, we used group-based trajectory modeling.^13^ Each individual was assigned into homogenous subgroups representing distinct trajectories of mean number sickness absence and disability pension days per year over the 4 years. Akaike information criterion and average posterior probability were used to determine the best-fitting model and group homogeneity. Each stroke patient was assigned to the trajectory group with highest probability of belonging. In these trajectory analyses, patients who deceased or emigrated during the study period were excluded. A lower cutoff at 10% group prevalence for each trajectory group was pre-determined. Multinomial regression analysis with reference to the trajectory group with persistently low sickness absence and/or disability pension was performed. Subsequently, crude and adjusted odds ratios (OR) and 95% confidence intervals (adjusted for sex, age, socio-demographic factors, hypertension, atrial fibrillation, and diabetes) for predictors for belonging to identified trajectory groups were computed for the stroke patient cohorts in 2000 and 2015.

Analyzes were performed in R (v 4.1.1). Packages crimCV and nnet were used for trajectory analysis and multinomial modeling.

The project was approved by the Regional Ethical Review Board in Stockholm, Sweden (diary number: 2007/762-31 and 2021/06441-02). Patient consent is not applicable in this type of study based on anonymized register data, thus waived by the Ethical Review Board.^14^

## Results

In the four cohorts of people aged 18–61 when having an incident ischemic stroke in 2000, 2005, 2010, or 2015, the number of patients were 2728, 2738, 2676, and 2531, respectively. Baseline characteristics in the calendar year before inclusion for the stroke patients and the reference individuals are presented in Table 1. Mean age for the incident stroke event remained unchanged over the study years (53 years), as did the proportion of men (64%).

Proportions of people with sickness absence and/or disability pension in each cohort is presented in Table 2. The rate of stroke patients with sickness absence in Y_-1_ ranged from 34.6% (2000) to 27.4% (2010) and corresponding rates among references from 16.3% (2000) to 9.9% (2010). Disability pension rates for stroke patients during Y_-1_ ranged from 34.0% (2005) to 20.5% (2015), and for references; from 18.4% (2005) to 10.6% (2015). When comparing the number of people with sickness absence and disability pension per year, stroke patients had significantly higher rates compared to references for all studied years. Mortality rate within the first year after stroke event decreased from 8.1% in 2000 to 4.8% in 2015.

**Table 2.** Numbers and proportion of people who had sickness absence (SA), disability pension (DP), emigrated, or died each year in relation to stroke date, among incident ischemic stroke patients, and among their matched references. *P*-values for stroke patient versus reference per year calculated by Chi-square test for SA and DP. Significant comparison (*p* < 0.0001) between stroke patients and references, per year denoted by ****.

Relative year	Y_-1_	Y_1_	Y_2_	Y_3_
Stroke patients 2000, *n* = 2728				
SA	944 (34.6)****	1661 (60.9)****	1104 (44.1)****	698 (28.6)****
DP	805 (29.5)****	971 (35.6)****	1246 (49.8)****	1451 (59.5)****
Emigrated	<10	<10	<10	<10
Death	N/A	220 (8.1)	63 (2.5)	60 (2.5)
References 2000, *n* = 13,640				
SA	2219 (16.3)	2356 (17.3)	2393 (17.7)	2225 (16.7)
DP	2112 (15.5)	2377 (17.4)	2646 (19.6)	2868 (21.5)
Emigrated	43 (0.3)	23 (0.2)	27 (0.2)	21 (0.2)
Death	N/A	108 (0.8)	93 (0.7)	78 (0.6)
Stroke patients 2005, *n* = 2738				
SA	867 (31.7)****	1575 (57.5)****	1005 (39.6)****	590 (23.8)****
DP	932 (34.0)****	1082 (39.5)****	1321 (52.0)****	1498 (60.5)****
Emigrated	<10	<10	<10	<10
Death	N/A	196 (7.2)	59 (2.3)	59 (2.4)
References 2005, *n* = 13,690				
SA	2061 (15.1)	1974 (14.4)	1803 (13.3)	1554 (11.6)
DP	2516 (18.4)	2716 (19.8)	2812 (20.7)	2922 (21.7)
Emigrated	29 (0.2)	37 (0.3)	29 (0.2)	31 (0.2)
Death	N/A	87 (0.6)	69 (0.5)	87 (0.6)
Stroke patients 2010, *n* = 2676				
SA	732 (27.4)****	1546 (57.8)****	900 (35.5)****	662 (26.4)****
DP	750 (28.0)****	776 (29.0)****	860 (33.9)****	1007 (40.2)****
Emigrated	17 (0.6)	<10	<10	<10
Death	N/A	136 (5.1)	31 (1.2)	43 (1.7)
References 2010, *n* = 13,380				
SA	1325 (9.9)	1337 (10.0)	1368 (10.3)	1409 (10.7)
DP	1884 (14.1)	1868 (14.0)	1830 (13.8)	1818 (13.8)
Emigrated	80 (0.6)	31 (0.2)	31 (0.2)	36 (0.3)
Death	N/A	71 (0.5)	65 (0.5)	61 (0.5)
Stroke patients 2015, *n* = 2531				
SA	772 (30.5)****	1665 (65.8)****	1035 (43.1)****	740 (31.5)****
DP	519 (20.5)****	570 (22.5)****	632 (26.3)****	708 (30.2)****
Emigrated	12 (0.5)	<10	<10	<10
Death	N/A	122 (4.8)	53 (2.2)	37 (1.6)
References 2015, *n* = 12,655				
SA	1588 (12.5)	1651 (13.0)	1626 (13.0)	1543 (12.4)
DP	1343 (10.6)	1381 (10.9)	1377 (11.0)	1382 (11.1)
Emigrated	90 (0.7)	33 (0.3)	35 (0.3)	36 (0.3)
Death	N/A	64 (0.5)	42 (0.3)	46 (0.4)

**Figure 1.**
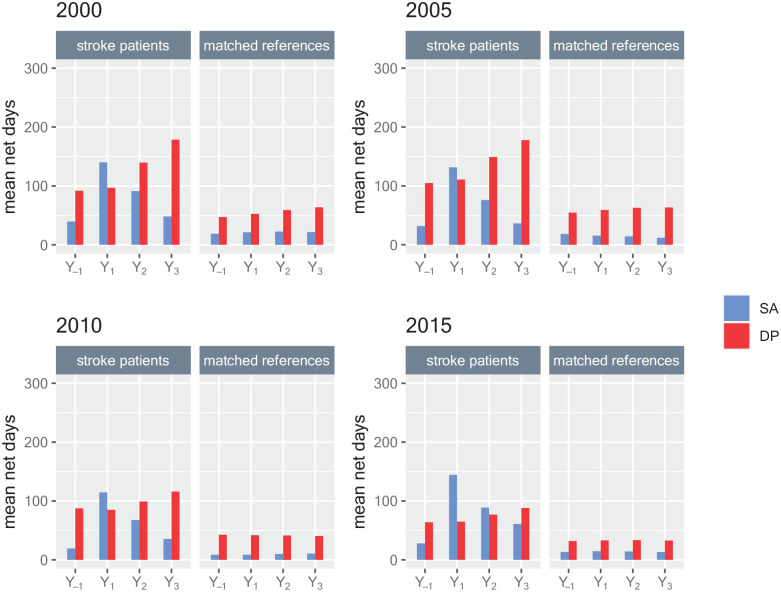
Mean annual sickness absence (SA) and disability pension (DP) net days for all incident ischemic stroke patients aged 18–61 in 2000, 2005, 2010, or 2015, respectively, and for matched references, regarding 1 year before the stroke date (Y_-1_) and the 3 years after the stroke date (Y_1_, Y_2_, Y_3_).

Figure 1 (and corresponding data in Supplemental Table S1) show mean annual days with sickness absence and disability pension, respectively, among all patients in the respective cohorts and their references. Among the stroke cohorts, mean sickness absence days were higher than among references; Y_1_ 2000: 140.10 (SD: 152.78) versus 21.23 (66.27); Y_1_ 2005: 131.50 (148.92) versus 15.53 (55.15); Y_1_ 2010: 114.53 (139.08) versus 8.83 (39.52); Y_1_ 2015: 144.32 (146.18) versus 14.73 (54.72). Their number of disability pension days was near the double compared to among references; Y_1_ 2000: 96.99 (151.13) versus 52.56 (122.02); Y_1_ 2005: 110.79 (157.35) versus 59.26 (127.60); Y_1_ 2010: 85.06 (146.47) versus 41.92 (110.57); Y_1_ 2015: 64.93 (132.17) versus 33.05 (99.60).

**Figure 2.**
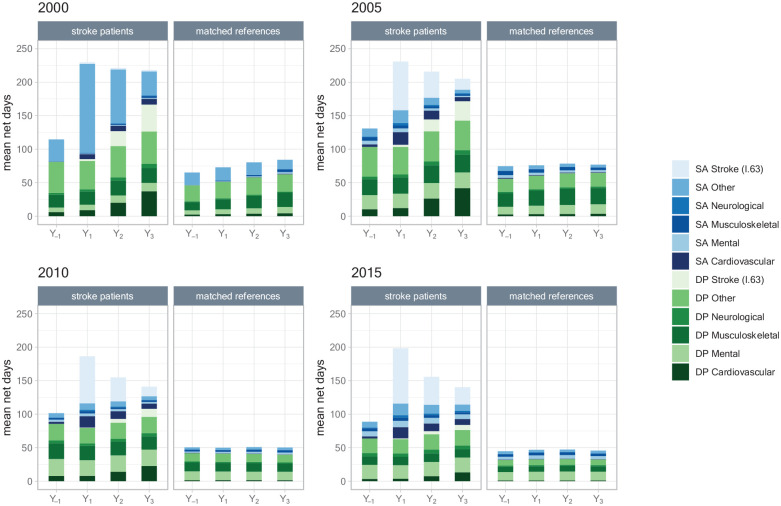
Mean annual net days of diagnoses-specific sickness absence (SA) and disability pension (DP) among patients with an incident ischemic stroke in 2000, 2005, 2010, or 2015, respectively, and among their matched references; in the year before stroke date (Y_-1_) and the 3 years after (Y_1_, Y_2_, Y_3_).

In Figure 2, mean annual number of diagnoses-specific sickness absence and disability pension days are presented for each of the four cohorts. Before 2005, diagnosis-specific coding for sickness absence was not mandatory, explaining the large number of days categorized as “Other.” Prior to the stroke event, sickness absence due to mental or musculoskeletal diagnoses were the most common, as they were in all years among the references.

**Figure 3.**
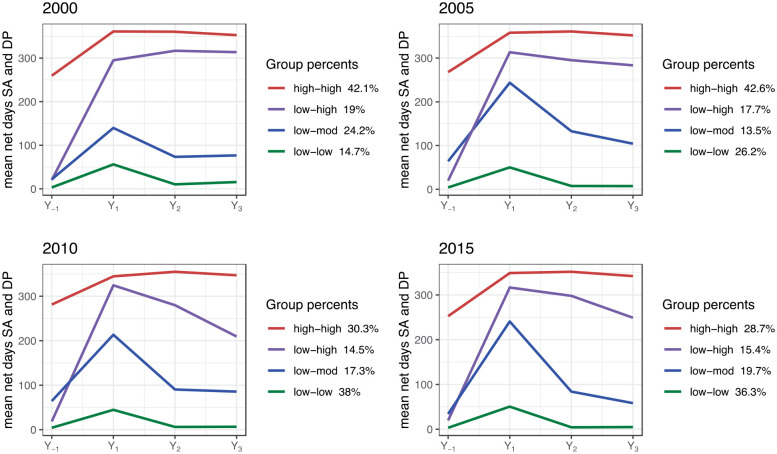
Trajectories of annual mean sickness absence and disability pension net days among the patients with an incident ischemic stroke in 2000, 2005, 2010, or 2015, respectively, in the year before the stroke date (Y_-1_) and in the 3 years following stroke date (Y_1_, Y_2_, Y_3_).

For each of the four stroke patient cohorts, we identified four different trajectories of sickness absence and disability pension days per year (named as in Figure 3). For the 2000, 2005, 2010, and 2015-cohorts, a total of 2369, 2407, 2440, and 2291 individuals were included in the trajectory analysis (i.e. lived in Sweden all 4 years). A distinct change in number of patients within different trajectories was seen as of 2010. In 2000 and 2005, the trajectory with high pre-stroke and high post-stroke trajectory group (high-high) comprised the largest patient group (42.1% and 42.6% of all). In 2010 and 2015, the largest trajectory group was instead those with low pre- and post-stroke numbers of mean days (low-low), 38% and 36.3%.

Factors associated with belonging to each trajectory group were computed for the first and last cohort (stroke incidence in 2000 or in 2015) (Table 3). For the 2000 cohort, adjusted OR for belonging to the high-high trajectory, compared to low-low trajectory, were elementary (OR 3.47; 95% CI (2.38–5.05)) and high school education level (2.12;(1.48–3.03)), being single (1.67;(1.29–2.16)), female sex (1.72;(1.31–2.26)), not born in Sweden (1.58;(1.08–2.30)), diabetes (1.86;(1.18–2.92)), and aged > 50 (2.25;(1.69–2.98)). For the 2015 cohort, with exception of born outside Sweden, the same factors were associated with belonging to the high-high trajectory.

**Table 3.** Crude and adjusted (adjusted for sex, age, education, marital status, family situation, living area, country of birth, hypertension, atrial fibrillation, and diabetes) odds ratios with 95% confidence intervals for belonging in trajectories low-high, low-mod, and high-high, respectively, related to the low-low trajectory (significant ORs in bold).

	Stroke event in 2000						Stroke event in 2015					
	Crude odds ratios			Adjusted odds ratios			Crude odds ratios			Adjusted odds ratios		
	low-high (purple)	low-mod (blue)	high-high (red)	low-high (purple)	low-mod (blue)	high-high (red)	low-high	low-mod	high-high	low-high	low-mod	high-high
Male sex	Reference											
Female sex	1.18 (0.87–1.60)	1.08 (0.81–1.44)	**1.60** (1.23–2.08)	1.24 (0.92–1.69)	1.10 (0.82–1.48)	**1.72** (1.31–2.26)	**1.37** (1.05–1.78)	**1.35** (1.06–1.72)	**1.85** (1.49–2.29)	**1.43** (1.10–1.87)	**1.36** (1.07–1.74)	**2.17** (1.74–2.72)
Age ⩽ 50	Reference											
Age > 50	1.31 (0.97–1.77)	1.30 (0.98–1.73)	**2.16** (1.64–2.83)	1.22 (0.89–1.66)	1.30 (0.97–1.74)	**2.25** (1.69–2.98)	1.19 (0.92–1.55)	0.93 (0.73–1.17)	**1.62** (1.30–2.03)	1.15 (0.87–1.51)	0.93 (0.73–1.19)	**1.63** (1.29–2.07)
University/college	Reference											
High school	1.02 (0.71–1.48)	0.91 (0.65–1.28)	**2.29** (1.61–3.24)	1.02 (0.70–1.48)	0.88 (0.63–1.24)	**2.12** (1.48–3.03)	**1.49** (1.11–2.00)	1.05 (0.81–1.35)	**2.27** (1.73–2.97)	**1.48** (1.10–2.00)	1.01 (0.78–1.31)	**2.19** (1.66–2.89)
Elementary	1.47 (0.99–2.16)	0.99 (0.68–1.42)	**3.90** (2.70–5.62)	1.43 (0.96–2.12)	0.94 (0.65–1.37)	**3.47** (2.38–5.05)	1.39 (0.96–2.02)	0.76 (0.54–1.08)	**3.62** (2.66–4.92)	1.43 (0.98–2.08)	0.79 (0.55–1.13)	**3.36** (2.44–4.62)
Married or civil partnership	Reference											
Neither married nor civil partnership	1.09 (0.82–1.45)	1.14 (0.87–1.49)	**1.57** (1.23–2.01)	1.11 (0.83–1.48)	1.18 (0.90–1.55)	**1.67** (1.29–2.16)	0.85 (0.67–1.10)	1.13 (0.90–1.43)	**1.54** (1.25–1.90)	0.84 (0.65–1.09)	1.12 (0.88–1.42)	**1.53** (1.23–1.90)
Born in Sweden	Reference											
Not born in Sweden	**1.53** (1.02–2.30)	1.04 (0.69–1.57)	**1.56** (1.09–2.25)	1.50 (0.99–2.28)	1.08 (0.71–1.63)	**1.58** (1.08–2.30)	0.85 (0.61–1.17)	**0.57** (0.41–0.79)	1.00 (0.78–1.29)	0.83 (0.60–1.16)	**0.57** (0.41–0.79)	0.96 (0.73–1.26)
Type of living area: cities	Reference											
Type of living area: towns and rural	0.89 (0.67–1.19)	1.07 (0.81–1.41)	1.23 (0.95–1.58)	0.95 (0.70–1.28)	1.11 (0.83–1.47)	1.23 (0.94–1.60)	1.07 (0.82–1.40)	0.99 (0.78–1.26)	1.16 (0.94–1.45)	1.01 (0.77–1.32)	0.96 (0.75–1.22)	1.11 (0.88–1.40)
No hypertension	Reference											
Hypertension	**1.51** (1.07–2.12)	1.06 (0.76–1.49)	0.92 (0.67–1.26)	1.43 (1.00–2.03)	1.01 (0.71–1.42)	0.84 (0.60–1.17)	1.10 (0.85–1.41)	1.09 (0.86–1.37)	1.09 (0.89–1.34)	1.03 (0.79–1.35)	1.10 (0.86–1.41)	0.92 (0.73–1.15)
No atrial fibrillation	Reference											
Atrial fibrillation	**4.90** (2.04–11.77)	**2.56** (1.04–6.31)	**2.60** (1.10–6.16)	**4.87** (2.02–11.74)	**2.53** (1.02–6.24)	2.38 (0.99–5.70)	1.21 (0.74–1.97)	1.11 (0.70–1.77)	1.26 (0.84–1.88)	1.20 (0.73–1.97)	1.18 (0.74–1.88)	1.17 (0.77–1.78)
No diabetes	Reference											
Diabetes	1.63 (1.00–2.67)	1.35 (0.83–2.19)	**1.97** (1.27–3.05)	1.47 (0.89–2.43)	1.34 (0.82–2.19)	**1.86** (1.18–2.92)	1.35 (0.93–1.97)	1.23 (0.86–1.76)	**2.02** (1.51–2.71)	1.36 (0.92–2.02)	1.35 (0.93–1.95)	**1.97** (1.43–2.69)

## Discussion

In this longitudinal population-based study, based on four cohorts from different time periods, ischemic stroke patients of working age, compared to matched references, already before the stroke had a higher level of both sickness absence and disability pension rates and days. Most such days were due to mental and musculoskeletal diagnoses. Moreover, we found a noteworthy decrease over the years 2000–2015 in mortality rate within the 12 months following stroke date.

This is the first study on sickness absence and disability pension comparing ischemic stroke patients to references, including all incident ischemic stroke patients of working age in a country. Similar results have been presented regarding stroke in general, however not specifically for ischemic stroke.^3–5^ We show that sickness absence and disability pension rates were nearly doubled compared to among matched references in the year before the ischemic stroke event. This finding could imply that additional factors other than those accounted for in this study contribute to sickness absence and disability pension before and after stroke. In the comparison between stroke patients and references regarding diagnosis-specific sickness absence in the year before the stroke event, most days were due to mental or musculoskeletal diagnoses, in agreement with a previous study not separating stroke subtypes.^4^ We found that the numbers of sickness absence days due to mental and musculoskeletal diagnoses in Y_-1_, were about the same in the first two cohorts, however, the number of days due to mental diagnoses increased in the two later cohorts compared to among references. Association between high levels of depression symptoms and risk of stroke have previously been shown.^15^ Here, we also found that mental disorders were so severe that they led to sickness absence. Ultimately, the mean sickness absence days was much lower than 365 days in all years following stroke event, indicating that in average the whole year was not spent absent from work. Around 60% of ischemic stroke patients were on SA in the year post-stroke, decreasing to around 40% in Y_2._ Although not the same measure, our results are in line with a previous study of return to work in Sweden showing that about 70% return within the first year post-stroke (ischemic and hemorrhagic).^16^

The observed decrease in mortality could be due to several factors. The introduction of reperfusion treatment in acute stroke treatment have affected outcome considerably in terms of stroke survival and functional dependence.^17^ During the study years, however, the use of thrombolysis (2000–2015; 0.6–12.0%) and mechanical thrombectomy (2015; 2%) was low.^18^ Admittance to stroke units is known to improve survival and outcome for stroke patients.^19^ In Sweden, the percentage of stroke patients admitted to stroke units increased from 73% in 2002 to 91% in 2015, which could have accounted for a large effect on survival. The decreased mortality rate might also account for our finding of higher sickness absence rates in 2015, that is, surviving stroke patients might have a higher risk of functional dependence affecting their work capacity.

That the number of disability pension days decreased over the study years is probably related to changed criteria for being granted disability pension in 2008, leading to that fewer were granted disability pension, and instead remained on long-term sickness absence.^20^ Moreover, who was granted disability pension instead of long-term sickness absence varied much between individuals, geographical areas, and over time.

Factors with high ORs for persistently high sickness absence and disability pension days per year were similar when comparing the 2000 with the 2015 cohort. A nationwide population-based study of stroke and ischemic heart disease found that age >50 years, female sex, lower educational level, single household, and diabetes were predictors for disability pension during the year following heart disease or stroke (ischemic and hemorrhagic).^3^ A study on sickness absence and disability pension in a subset of ischemic stroke patients found that male sex and higher income predicted more such days.^1^ However, this was another type of analyses than we performed, in a limited subset of the stroke population in Sweden.

The main strength of this study is the longitudinal cohort study design, that all patients in working ages with ischemic stroke from four different periods were included, enabling the possibility to handle both period and cohort effects, for example, regarding progress in stroke care. Most of the few previous studies on sickness absence and disability pension among stroke patients have not differed between stroke subtypes. In contrast, we focused on ischemic stroke patients, providing more clinical relevance. Additional strengths are no drop-outs, that all data were of administrative nature from high-quality nationwide registers,^21,22^ that is, no self-reports affected by recall bias, and the large numbers included, allowing for sub-group analyses. Other strengths are that information on sickness absence and disability pension concerned the years (365 days) before and after the stroke date, that is, was not based on calendar year, that we could calculate net days, and that sickness absence and disability pension days could be combined.

Limitations include lack of data on stroke severity and etiology. Moreover, we did not have data on functional dependence after stroke. Another limitation is that sickness absence spells with a duration of<15 days could not be included. However, that was the situation for both stroke patients and references, and for all studied years.

Results can be considered generalizable to other welfare states with similar availability to healthcare and sickness absence and disability pension security systems.

## Conclusion

Our nationwide study shows that incident ischemic stroke patients of working ages had higher rates of sickness absence and disability pension both before and after stroke compared to references matched on sociodemographic variables.

## Supplemental Material

sj-docx-1-eso-10.1177_23969873241261011 – Supplemental material for Sickness absence and disability pension patterns before and after ischemic stroke: A Swedish longitudinal cohort study with matched references Supplemental material, sj-docx-1-eso-10.1177_23969873241261011 for Sickness absence and disability pension patterns before and after ischemic stroke: A Swedish longitudinal cohort study with matched references by Mihae Roland, Ann-Sofie Rudberg, Kristina Alexanderson and Christina Sjöstrand in European Stroke Journal

sj-docx-2-eso-10.1177_23969873241261011 – Supplemental material for Sickness absence and disability pension patterns before and after ischemic stroke: A Swedish longitudinal cohort study with matched references Supplemental material, sj-docx-2-eso-10.1177_23969873241261011 for Sickness absence and disability pension patterns before and after ischemic stroke: A Swedish longitudinal cohort study with matched references by Mihae Roland, Ann-Sofie Rudberg, Kristina Alexanderson and Christina Sjöstrand in European Stroke Journal
