# Supplementary material for: Sickness absence and disability pension patterns before and after ischemic stroke: A Swedish longitudinal cohort study with matched references
Source: Eur Stroke J. 2024 Jun 14;10(1):268–77. doi: 10.1177/23969873241261011 (PMC11569591; doi:10.1177/23969873241261011)
Supplement: sj-docx-1-eso-10.1177_23969873241261011 – Supplemental material for Sickness absence and disability pension patterns before and after ischemic stroke: A Swedish longitudinal cohort study with matched references [file sj-docx-1-eso-10.1177_23969873241261011.docx]

STROBE Statement—checklist of items that should be included in reports of observational studies

|  | Item No. | Recommendation | Page  No. | Relevant text from manuscript |
| --- | --- | --- | --- | --- |
| **Title and abstract** | 1 | (*a*) Indicate the study’s design with a commonly used term in the title or the abstract | 1 | See abstract |
|  |  | (*b*) Provide in the abstract an informative and balanced summary of what was done and what was found | 1 | See abstract |
| Introduction | | | |  |
| Background/rationale | 2 | Explain the scientific background and rationale for the investigation being reported | 2 | “only one study (N=6637), separated stroke of ischemic and hemorrhagic origin …” “Stroke affects populations with low socioeconomic status disproportionally …. motivating the use of matched references from the general population.” |
| Objectives | 3 | State specific objectives, including any prespecified hypotheses | 2 | “The main aim was to determine sickness absence and disability pension rates and net days before and after ischemic stroke, as well as to compare such rates and days to those among matched references from the general population.” |
| Methods | | | |  |
| Study design | 4 | Present key elements of study design early in the paper | 2 | “Four cohorts of incident ischemic stroke patients and matched references were studied, each cohort was followed over four years..” |
| Setting | 5 | Describe the setting, locations, and relevant dates, including periods of recruitment, exposure, follow-up, and data collection | 3-4 | See heading “study population and data sources” |
| Participants | 6 | (*a*) *Cohort study*—Give the eligibility criteria, and the sources and methods of selection of participants. Describe methods of follow-up  *Case-control study*—Give the eligibility criteria, and the sources and methods of case ascertainment and control selection. Give the rationale for the choice of cases and controls  *Cross-sectional study*—Give the eligibility criteria, and the sources and methods of selection of participants | 4 | Cohort study: “We included all people living in Sweden admitted to hospital during the respective index year for a first ischemic stroke diagnosis (ICD-10: I63:0-9) as main diagnosis, when aged 18-61 years….”. This was done for four different cohorts from four different time periods. |
|  |  | (*b*) *Cohort study*—For matched studies, give matching criteria and number of exposed and unexposed  *Case-control study*—For matched studies, give matching criteria and the number of controls per case | 4 | Cohort study “For each included stroke patient, we included five reference individuals. They were randomly chosen among the population of Sweden with no prior stroke, and were matched on index year and the parameters; age, sex, birth country, educational level, and type of living area.“ Between 2500-2800 patients per cohort plus references for all the four cohorts |
| Variables | 7 | Clearly define all outcomes, exposures, predictors, potential confounders, and effect modifiers. Give diagnostic criteria, if applicable | 3-5 | “… mean annual sickness absence/disability pension days among all individuals in the cohort …”, “adjusted for sex, age, socio-demographic factors, hypertension, atrial fibrillation, and diabetes” |
| Data sources/ measurement | 8* | For each variable of interest, give sources of data and details of methods of assessment (measurement). Describe comparability of assessment methods if there is more than one group | 3-4 | See header “Study population and data sources” |
| Bias | 9 | Describe any efforts to address potential sources of bias | 3-5 | Using register-based data, not self-reports, and no loss to follow up. Including all patients in a country who had on incident stroke, Five reference individuals per patient. They were randomly chosen among the population of Sweden”, “we did not include information on sickness absence spells that were shorter than 15 days, in order not to introduce bias regarding unemployed or self-employed.” |
| Study size | 10 | Explain how the study size was arrived at | 4 | “We included all people living in Sweden admitted to hospital during the respective index year for a first ischemic stroke diagnosis (ICD-10: I63:0-9) as main diagnosis, when aged 18-61 years.” |

Continued on next page

| Quantitative variables | 11 | Explain how quantitative variables were handled in the analyses. If applicable, describe which groupings were chosen and why | 5 | See header statistical analysis |
| --- | --- | --- | --- | --- |
| Statistical methods | 12 | (*a*) Describe all statistical methods, including those used to control for confounding | 5 | See header statistical analysis |
|  |  | (*b*) Describe any methods used to examine subgroups and interactions | 5 | See header statistical analysis |
|  |  | (*c*) Explain how missing data were addressed | 3  and  Table 1 | There were very few people with missing data. “Data was missing on birth country for one individual in the 2005 cohort and two individuals in 2010; those were categorized as ‘Rest of the World’. Information on educational level was missing for 199 (1.5%), 175 (1.1%), 165 (1.0%), 171 (1.1%) patients in the 2000, 2005, 2010, and 2015-cohorts, respectively; those were categorized as having the lowest educational level.” |
|  |  | (*d*) *Cohort study*—If applicable, explain how loss to follow-up was addressed  *Case-control study*—If applicable, explain how matching of cases and controls was addressed  *Cross-sectional study*—If applicable, describe analytical methods taking account of sampling strategy | 3-5 | There were no loss to follow-up in the data. We had data on all in the four cohorts and could follow them up till end of follow up or until they emigrated or died – these events we had information about. Those who died or emigrated during the follow-up were excluded in the trajectory analyses. |
|  |  | (*e*) Describe any sensitivity analyses | NA |  |
| Results | | | | |
| Participants | 13* | (a) Report numbers of individuals at each stage of study—eg numbers potentially eligible, examined for eligibility, confirmed eligible, included in the study, completing follow-up, and analysed |  | Table 1 |
|  |  | (b) Give reasons for non-participation at each stage | 3-4 | Patients who deceased or emigrated during the follow-up period were excluded from one type of analyses, namely trajectory analyses. |
|  |  | (c) Consider use of a flow diagram | NA |  |
| Descriptive data | 14* | (a) Give characteristics of study participants (eg demographic, clinical, social) and information on exposures and potential confounders |  | Table 1 |
|  |  | (b) Indicate number of participants with missing data for each variable of interest | 3-4, Table 1 | Data was missing for some people regarding birth country and educational level. Information on numbers and how they were categorized is given in the method section and in Table 1 |
|  |  | (c) *Cohort study*—Summarise follow-up time (eg, average and total amount) | 3-5 | Each patient and matched reference person were followed from one year (365 days) prior the date of the patient’s stroke event (Y_-1_) through three years after the stroke event date (Y_1_, Y_2_, Y_3_)”. |
| Outcome data | 15* | *Cohort study*—Report numbers of outcome events or summary measures over time |  | Table 2 |
|  |  | *Case-control study—*Report numbers in each exposure category, or summary measures of exposure |  |  |
|  |  | *Cross-sectional study—*Report numbers of outcome events or summary measures |  |  |
| Main results | 16 | (*a*) Give unadjusted estimates and, if applicable, confounder-adjusted estimates and their precision (eg, 95% confidence interval). Make clear which confounders were adjusted for and why they were included |  | Table 3 |
|  |  | (*b*) Report category boundaries when continuous variables were categorized |  | Table 3 |
|  |  | (*c*) If relevant, consider translating estimates of relative risk into absolute risk for a meaningful time period |  | NA |

Continued on next page

| Other analyses | 17 | Report other analyses done—eg analyses of subgroups and interactions, and sensitivity analyses | NA | Table 3 |
| --- | --- | --- | --- | --- |
| Discussion | | | | |
| Key results | 18 | Summarise key results with reference to study objectives | 8 | “ischemic stroke patients of working age, compared to matched references, already before the stroke had a higher level of both sickness absence and disability pension rates and days..” |
| Limitations | 19 | Discuss limitations of the study, taking into account sources of potential bias or imprecision. Discuss both direction and magnitude of any potential bias | 10 |  |
| Interpretation | 20 | Give a cautious overall interpretation of results considering objectives, limitations, multiplicity of analyses, results from similar studies, and other relevant evidence | 11 | See header conclusion |
| Generalisability | 21 | Discuss the generalisability (external validity) of the study results | 11 | “Results can be generalizable..” |
| Other information | |  | | |
| Funding | 22 | Give the source of funding and the role of the funders for the present study and, if applicable, for the original study on which the present article is based | 11 | See Funding |

*Give information separately for cases and controls in case-control studies and, if applicable, for exposed and unexposed groups in cohort and cross-sectional studies.

**Note:** An Explanation and Elaboration article discusses each checklist item and gives methodological background and published examples of transparent reporting. The STROBE checklist is best used in conjunction with this article (freely available on the Web sites of PLoS Medicine at http://www.plosmedicine.org/, Annals of Internal Medicine at http://www.annals.org/, and Epidemiology at http://www.epidem.com/). Information on the STROBE Initiative is available at www.strobe-statement.org.
