# Supplementary material for: Sickness absence and disability pension patterns before and after ischemic stroke: A Swedish longitudinal cohort study with matched references
Source: Eur Stroke J. 2024 Jun 14;10(1):268–77. doi: 10.1177/23969873241261011 (PMC11569591; doi:10.1177/23969873241261011)
Supplement: sj-docx-2-eso-10.1177_23969873241261011 – Supplemental material for Sickness absence and disability pension patterns before and after ischemic stroke: A Swedish longitudinal cohort study with matched references [file sj-docx-2-eso-10.1177_23969873241261011.docx]

Supplementary material

| **Relative year** | **Y-1** | **Y1** | **Y2** | **Y3** |
| --- | --- | --- | --- | --- |
| Stroke patients 2000, n=2728 | | | | |
| SA (mean (SD)) | 39.59 (91.12) | 140.10 (152.78) | 91.21 (131.79) | 48.15 (101.09) |
| DP (mean (SD)) | 91.74 (151.19) | 96.99 (151.13) | 139.75 (163.45) | 178.58 (167.93) |
| References 2000, n=13640 | | | | |
| SA (mean (SD)) | 18.89 (61.96) | 21.23 (66.27) | 22.57 (69.08) | 21.70 (68.52) |
| DP (mean (SD)) | 47.09 (116.57) | 52.56 (122.02) | 58.96 (127.84) | 63.77 (130.79) |
| Stroke patients 2005, n=2738 | | | | |
| SA (mean (SD)) | 31.95 (80.22) | 131.50 (148.92) | 76.13 (120.08) | 36.29 (87.63) |
| DP (mean (SD)) | 104.74 (156.95) | 110.79 (157.35) | 149.35 (164.70) | 177.89 (166.84) |
| References 2005, n=13690 | | | | |
| SA (mean (SD)) | 18.38 (61.01) | 15.53 (55.15) | 14.27 (53.29) | 12.00 (48.00) |
| DP (mean (SD)) | 54.73 (123.57) | 59.26 (127.60) | 62.78 (130.69) | 63.51 (129.66) |
| Stroke patients 2010, n=2676 | | | | |
| SA (mean (SD)) | 19.41 (58.21) | 114.53 (139.08) | 67.73 (119.55) | 35.47 (79.11) |
| DP (mean (SD)) | 87.48 (148.77) | 85.06 (146.47) | 99.05 (152.56) | 116.14 (157.38) |
| References 2010, n=13380 | | | | |
| SA (mean (SD)) | 8.52 (38.32) | 8.83 (39.52) | 10.14 (43.93) | 10.62 (45.24) |
| DP (mean (SD)) | 42.68 (111.57) | 41.92 (110.57) | 41.44 (109.98) | 40.43 (107.95) |
| Stroke patients 2015, n=2531 | | | | |
| SA (mean (SD)) | 28.03 (75.08) | 144.32 (146.18) | 88.70 (133.91) | 60.95 (115.91) |
| DP (mean (SD)) | 63.86 (132.72) | 64.93 (132.17) | 76.80 (139.72) | 88.05 (146.76) |
| Reference 2015, n=12655 | | | | |
| SA (mean (SD)) | 13.46 (51.04) | 14.73 (54.72) | 14.34 (54.10) | 13.23 (51.27) |
| DP (mean (SD)) | 31.99 (98.19) | 33.05 (99.60) | 33.54 (100.56) | 32.80 (98.81) |

**Table S1.** Mean annual sickness absence and disability pension net days among ischemic stroke patients and their references, in the year before the stroke date (Y-1) and in the three years following stroke date (Y1, Y2, Y3), by their respective inclusion year.
